# Islet Autoimmunity Identifies a Unique Pattern of Impaired Pancreatic Beta-Cell Function, Markedly Reduced Pancreatic Beta Cell Mass and Insulin Resistance in Clinically Diagnosed Type 2 Diabetes

**DOI:** 10.1371/journal.pone.0106537

**Published:** 2014-09-16

**Authors:** Angela Subauste, Roberto Gianani, Annette M. Chang, Cynthia Plunkett, Susan L. Pietropaolo, Ying-Jian Zhang, Emma Barinas-Mitchell, Lewis H. Kuller, Andrzej Galecki, Jeffrey B. Halter, Massimo Pietropaolo

**Affiliations:** 1 Division of Diabetes, Endocrinology and Metabolism, Department of Medicine, Baylor College of Medicine, Houston, Texas, United States of America; 2 The Brehm Center for Diabetes Research, Division of Metabolism, Endocrinology & Diabetes, Department of Internal Medicine, University of Michigan Medical School, Ann Arbor, Michigan, United States of America; 3 Geriatrics Center and Institute of Gerontology, Department of Internal Medicine, University of Michigan Medical School, Ann Arbor, Michigan, United States of America; 4 Department of Epidemiology, Graduate School of Public Health, University of Pittsburgh, Pittsburgh, Pennsylvania, United States of America; 5 Division of Endocrinology, Department of Medicine, University of Mississippi Medical Center, Jackson, Mississippi, United States of America; University of Cincinnati College of Medicine, United States of America

## Abstract

There is a paucity of literature describing metabolic and histological data in adult-onset autoimmune diabetes. This subgroup of diabetes mellitus affects at least 5% of clinically diagnosed type 2 diabetic patients (T2DM) and it is termed Latent Autoimmune Diabetes in Adults (LADA). We evaluated indexes of insulin secretion, metabolic assessment, and pancreatic pathology in clinically diagnosed T2DM patients with and without the presence of humoral islet autoimmunity (Ab). A total of 18 patients with at least 5-year duration of clinically diagnosed T2DM were evaluated in this study. In those subjects we assessed acute insulin responses to arginine, a glucose clamp study, whole-body fat mass and fat-free mass. We have also analyzed the pancreatic pathology of 15 T2DM and 43 control cadaveric donors, using pancreatic tissue obtained from all the T2DM organ donors available from the nPOD network through December 31, 2013. The presence of islet Ab correlated with severely impaired β-cell function as demonstrated by remarkably low acute insulin response to arginine (AIR) when compared to that of the Ab negative group. Glucose clamp studies indicated that both Ab positive and Ab negative patients exhibited peripheral insulin resistance in a similar fashion. Pathology data from T2DM donors with Ab or the autoimmune diabetes associated DR3/DR4 allelic class II combination showed reduction in beta cell mass as well as presence of autoimmune-associated pattern A pathology in subjects with either islet autoantibodies or the DR3/DR4 genotype. In conclusion, we provide compelling evidence indicating that islet Ab positive long-term T2DM patients exhibit profound impairment of insulin secretion as well as reduced beta cell mass seemingly determined by an immune-mediated injury of pancreatic β-cells. Deciphering the mechanisms underlying beta cell destruction in this subset of diabetic patients may lead to the development of novel immunologic therapies aimed at halting the disease progression in its early stage.

## Introduction

Type 2 diabetes mellitus (T2DM) represents a group of heterogeneous metabolic diseases encompassing a wide variety of pathogenetic mechanisms ranging from abnormalities related to glucose-stimulated insulin secretion [1], [2], insulin resistance [3], [4], endoplasmic reticulum stress-induced β cell apoptosis [5] and inflammatory-mediated lesions of the target organ [6]–[10]. Previous studies have shown that 5–10% of patients with T2DM exhibit autoantibodies against the islet antigen glutamic acid decarboxylase, 65 kDa isoform, (GAD), one of the well-recognized markers of islet cell autoimmunity in Type 1 diabetes (T1DM). This subgroup of diabetes mellitus affects at least 5% of clinically diagnosed type 2 diabetic patients (T2DM) [11]–[16]. These patients are commonly classified as having Latent Autoimmune Diabetes in Adults (LADA) [17]–[20]. This group is characterized by progression to insulin-requiring diabetes, lower C-peptide levels and usually lower body mass index (BMI) in younger subjects (e.g. 45 years of age) [12], [19], [21].

Whether LADA represents a distinct group from T1DM or it is just the same entity presenting later in life is still under debate. Recent literature suggests this group is distinct from T1DM genetically and immunologically [22]. Detailed metabolic and histologic studies in LADA patients are still lacking. We have previously reported two heterogeneous patterns of beta cell pathology in Diabetes Mellitus namely pattern A (characterized by the presence of pseudo-atrophic islets, i.e. islets completely devoid of insulin positive cells) and pattern B (characterized by the absence of pseudo-atrophic islets) [23]. Pattern A is strongly associates with autoimmune Type 1 diabetes (T1a) while pattern B is usually seen in non-autoimmune Type 1 diabetes (T1b).

In this study we evaluated patients affected by LADA and compared to antibody negative T2DM of comparable BMI, age and duration of diabetes. For the first time, our analysis included beta cell assessment through arginine stimulation and peripheral insulin sensitivity through a euglycemic clamp in addition to the assessment of the pathology from T2DM adult onset organ donors with and without islet autoimmunity from the unique nPOD collection. Our observations demonstrate that T2DM patients with evidence for islet autoimmunity exhibit unique metabolic and histological characteristics including insulin deficiency and pattern A pancreatic pathology.

## Material and Methods

### Human Subjects

Type 2 diabetic patients were recruited in the Metabolism, Endocrinology & Diabetes (MEND) clinic at University of Michigan. The protocol was approved by the University of Michigan Institutional Review Board. Participants provided their written informed consent to participate in this study. A total of 18 subjects with clinically diagnosed T2DM participated in this study. Seventeen out of 18 subjects were of Caucasian and one of African American descent. Inclusion criteria included: 40 years of age or older, type 2 was diagnosed according to standard National Diabetes Data Group criteria [24].

The participants recruited in the present study were treated with insulin, metformin or a combination of both. Patients on insulin had duration of clinically diagnosed T2DM for at least 5 years. Exclusion criteria included: Onset of diabetes before the age of 40, first degree relatives of T1DM patients, pregnancy, concurrent illness and/or disease that limits life expectance or lead to immunosuppressive or immunomodulatory therapy during the time of the study, history of liver disease, substance abuse and deemed unlikely to comply with the protocol.

Participants were categorized in the following three groups: antibody negative clinically diagnosed T2DM treated with metformin only, antibody negative on insulin±metformin, antibody positive on insulin±metformin. In terms of therapy, 2 of the 6 subjects in the antibody negative group on insulin were also treated with metformin. In the antibody positive insulin-dependent group, 3 of 7 were also treated with metformin. All of the subjects who were treated with insulin at the time of testing used at least 6 months of oral hypoglycemic agents prior to transitioning to insulin therapy. All participants were evaluated for the presence of GAD65, IA-2 and ZnT8 antibodies. Six out of those 18 participants were positive for GAD65 antibodies: Of those GAD65 Ab positive patients, 2 were also positive for IA-2 and 3 for ZnT8 antibodies. One of these subjects was positive for IA-2 Ab alone.

As shown in Table 1, there was no significant difference in terms of age, body mass index (BMI) or HbA1c level between the three groups. In terms of HbA1c level, the majority of the subjects were in the 6-8% range, except for one participant in the antibody positive group (HbA1c of 11.4%). The best glycemic control trend was seen in the metformin only group both in terms of HbA1c (HbA1c 7.1% vs. 7.6% for the antibody negative on insulin group) and in terms of the fasting glucose (133 vs.175 mg/dl). The metformin only group had a statistically significant shorter duration of diabetes (3.8 vs. 16.2 years for the antibody negative group on insulin therapy, which has the longest duration of diabetes).

**Table 1:** Clinical characteristics. All of the blood sampling was performed under fasting conditions.

	Antibody negative (metformin)	Antibody negative (insulin ± metformin)	Antibody positive** (insulin ± metformin)
**Age (years)**	52.87 (42–68)	55.8 (42–69)	55 (42–68)
**Gender (M/F)**	2/3	2/4	3/4
**Diabetes duration (years)**	3.8 (2–6)	16.2 (7–21)	12.8 (6.5–20)
**Metformin use**	5/5	2/6	3/7
**HbA1c (%)**	7.1 (6.5–7.5)	7.6 (6.2–9.5)	8.5 (7–11.4)
**Weight (kg)**	77.2 (69.4–86.6)	87.6 (76.7–103.4)	81.6 (63.8-99)
**BMI (kg/m^2^)**	27.6 (26–29.6)	31.7 (25.5–36.2)	32 (26.9–47)
**Fat (%)**	35.2 (29.8–39.7)	36.7 (18–46.7)	35.7 (24.9–47.6)
**Central fat (g)**	2853 (2300–3168)	3580 (1538–5317)	2933 (1875–4027)
**Central fat (%)**	11.1 (8.5–12.7)	12.3 (9.4–13.5)	10.9 (8.5–14.2)
**HDL (mg/dL)**	54.7 (45.8–62)	41 (30–45) *	74 (56–119) *
**TG (mg/dL)**	87.2 (38–137)	107 (57–144)	62 (31–163)
**FFA (mg/dL)**	0.93 (0.78–1.00)	1.0 (0.47–1.44)	0.7 (0.36–0.88)
**Glucose (mg/dL)**	133 (97–161)	175 (93–234)	204 (121–276)
**C-peptide (ng/mL)**	2 (1.3–3.5)	2.73 (1.4–4.4) *	0.45 (0–0.6) *
**Pro-insulin (pmol/L)**	19 (9.5–26)	32.17 (13–95) *	9.2 (0–19) *
**Adiponectin (ng/mL)**	7944 (4818–12848)	8819 (4295–12410)	15877 (7380–22118)
**CRP (mg/L)**	1.9 (0.7–3.4)	2.3(0.5–4.5)	3.45 (0.2–8.7)

* = p<0.05 (antibody negative (insulin± metformin) vs antibody positive (insulin± metformin)).

**Antibody Positive Group: 6 out of 7 patients were positive for GAD65 Ab. Of those 6 GAD65 Ab positives, 2 were also positive for IA-2 and 3 for ZnT8 Ab. One of these subjects was positive for IA-2 Ab alone. Antibody titer: 3 GAD65 Ab positive 3^rd^ tertile; 2 IA-2 Ab positive 3^rd^ tertile.

### Organ donors

Organ donors were procured through the JDRF sponsored network of pancreatic organ donors with diabetes (nPOD) initiative [25]. Pancreatic specimens were obtained from all the donors, originally diagnosed with T2DM, available from nPOD through December 31, 2013 [23], [25]. Pancreatic tissue sections were prepared as previously described [23]. For this study, we analyzed the pancreatic pathology of 15 T2DM and 43 controls. This group consisted of all T2DM donors (not treated with incretins) and all control donors (with pancreatic weight data) available in the nPOD collection as 12/31/2013. Three of the T2DM donors were African American, one was Asian-American, eight were Caucasian and three were Hispanic. The mean age of onset in this group was 32 years (median 26.3 years, range 15.8 to 60.3 years), while the mean age at death was 42.8 years (median 42.8 years, range = 18.8 to 76.3 years) (Table 2 summarizes the demographic and immunological features of the T2DM antibody positive donors).

**Table 2:** Demographic and immunological characteristics of clinically diagnosed T2DM cadaveric donors captured through the nPOD network.

nPOD ID #	Gender	Age at death (years)	DM duration (years)	Ethnicity	AA status	HLA DR
**6028**	M	33.2	17.0	AA	N	DR6;DR6
**6037**	M	76.3	50.0	CA	N	DR4;DR3
**6059**	M	18.8	0.2	HI	N	DR7;DR8
**6108**	M	57.9	2.0	ASA	N	DR15;DR15
**6110**	F	20.7	0.0	AA	N	DR3;DR2
**6114**	M	42.8	2.0	CA	N	DR7;DR2
**6124**	F	63.3	3.0	CA	N	DR4;DR2
**6127**	F	44.8	10.0	CA	N	DR4;DR3
**6133**	F	45.80	20.0	CA	N	DR10;DR2
**6139**	F	37.2	1.5	HI	N	DR4;DR2
**6142**	F	29.8	14.2	HI	P	DR6;DR1
**6149**	F	39.3	16.0	AA	P	DR9;DR2
**6175**	M	42.9	12.0	CA	N	DR3;DR3
**6191**	F	62.0	10.0	CA	N	DR4;DR7
**6203**	M	27.6	5.0	CA	N	DR11;DR11

AA: African American donors, AS: Asian-American donors, CA: Caucasian donors, HI: Hispanic donors.

The control group was composed of five African-American, thirty- five Caucasian and three Hispanic donors. Twenty-five of these donors were male and thirteen were female. The mean age at death in this group was 28.9 years (median 22.1 years, range 2.2 – 45.9).

### Islet Autoantibody testing

Serum samples were assayed for autoantibodies directed to GAD65, IA-2 and ZnT8 as previously described (15, 16). The GAD65 construct was donated by Dr. Lernmark, while the IA-2 construct (ICA512bdc amino acid 267–556; 630–979) was donated by Dr. Eisenbarth. The results were expressed as an index (index = [cpm – negative control cpm]/[positive control cpm – negative control cpm]). Previously evaluated serum samples which were highly reactive for either IA-2 or GAD65 were utilized as positive controls, while pooled sera from non-diabetic individuals was utilized as a negative experimental control.

Additional IA-2 constructs were utilized in the detection of IA-2 autoantibodies. Dr. E. Bonifacio donated the IA-2ic construct (amino acids 601-979), while a full length IA-2 construct was also donated by Dr. Eisenbarth (amino acids 1–979). The ZnT8 construct was kindly donated by Dr. Hutton. GAD65 and IA-2 assays have been utilized repeatedly in proficiency workshops. Proficiency workshop results organized by the University of Florida, Gainesville (1995, 1996 and 1997), and the Diabetes Autoantibody Standardization Program (DASP, 2000, 2003, 2005, 2007, 2010), organized by WHO were as follows: 76–100% sensitivity, 90–100% specificity (100% specificity 3 times), and 100% validity for GAD65 and ICA512bdc autoantibodies; 48–84% sensitivity, 98–100% specificity, 87.5% validity and 91.6% consistency in the 1996, 2000 and 2003, 2005, 2007 and 2010. IA-2 full length and IA-2ic autoantibodies achieved ratings of 68%, 66% sensitivity and 98%, 99% specificity respectively in the 2007 DASP workshop.

### HLA Genotyping

HLA DR typing of organ donors was kindly provided by nPOD as previously reported [23], [26].

### Quantification of pancreatic beta cell mass

For each case, pancreatic beta cell mass (BCM) was quantified using computer assisted morphometric analysis of all available insulin stained sections from the head, body and tail of the pancreas. Briefly the insulin stained area was quantified with ImageProplus (Media Cybernetics, Rockville, MD) and expressed as ratio to the total pancreatic area. The average ratio between insulin stained area and total areas was multiplied by the pancreatic weight in gms thus yielding the estimated BCM in gms. The mean BCM of controls was utilized to expressed the BCM of each case in relation to controls with the formula: BCM case = (estimated BCM case/mean estimated BCM of controls) multiplied by 100.

### Islet function testing

The response to arginine, a potent insulin secretagogue, was utilized to evaluate β-cell function, according to well-established protocols [2]. Briefly, islet function was evaluated by measurement of acute insulin responses (AIR) to arginine at three glycemic levels (150, 250 and 500 mg/dL). After baseline samples were obtained for measurement of glucose, C-peptide, proinsulin, the acute insulin response to a maximally stimulating dose of arginine HCl (10% Arginine Hydrochloride, Pfizer, Lake Forest, IL) was measured. These responses were calculated from samples drawn 2, 3, 4, 5, 7 and 10 min. after this dose and all subsequent doses of arginine. Next, a variable rate infusion of 20% dextrose was administered to raise and maintain the plasma glucose level. Thirty minutes after the glucose clamp begins, prestimulus samples were again obtained and AIRs to a pulse of arginine was measured. The AIR to arginine at the highest glucose level (500 mg/dL) was defined as AIR_max_.

The C-peptide area under the curve (AUC) over the 10-min interval was estimated for each subject according to previously published protocols [27]. Disposition index (DI) is calculated as glucose infusion rate divided by the measured mean insulin concentration during the second hour of the clamp during the glucose clamp times AIR_max_. DI provides an indirect assessment of whether insulin secretion is appropriate for the level of insulin resistance (β-cell compensation for insulin resistance or β-cell function) [28].

### Glucose Clamp Studies

Glucose clamp studies were performed and this is regarded as a methodology for the determination of insulin sensitivity (18). Volunteers held metformin and long acting insulin 48 hours and 24 hours prior to the admission respectively. The glucose clamp study was started following administration of insulin bolus of 240 milliunits/m^2^/min for 5 minutes followed by insulin infusion of 80 milliunits/m^2^/min.

Plasma glucose levels were measured at 5 minute intervals and maintained at a target value of 90 mg/dl by an adjustable infusion of 20% dextrose, and the exact concentration of the infusion was measured prior to each study. Glucose infusion rates (GIR) were recorded at 5 minute intervals. Once steady-state conditions were achieved, rates of exogenous glucose infusion, equal rates of glucose disposal. Thus, GIR provides a quantitative measure of insulin-stimulated glucose metabolism. Plasma samples to determine glucose and insulin concentrations were drawn at *t* = −5, 0, 60, 120, 170, 180 min. At *t* = 180 min, all infusions were stopped with the exception of the 20% glucose. Plasma glucose was checked for at least 30 min. after glucose infusion was stopped.

### Body Composition Studies

Whole-body fat mass (FM) and fat-free mass (FFM) was assessed by Dual-energy X-ray Absorptiometry (DEXA) according to standard protocols [29]. Standing height and weight was checked in each participant, and the BMI was calculated as weight (kg)/height (m^2^). Additional image analysis was done for regional analysis of body composition.

### Statistical Analysis

Non-parametric Friedman test was used to compare acute insulin response. *p* values<0.05 were deemed statistically significant. The chi square test was utilized to compare proportions and determine a statistically significant association between two variables. Fisher's exact test was applied if any expected cell value in a 2×2 table was less than 5. Wilcoxon scores (rank sums) were applied for variable C-peptide AUC during the arginine test. Non-parameteric Friedman test was used for the comparisons of AIR among the Ab +, Ab- on insulin, Ab- on metformin groups. DI and GIR values between groups were compared using the Mann-Whitney test. Pearson Correlation was used to determine the degree of correlation between two variables. Data were analyzed using SAS 9.2 (SAS Institute Inc. Cary, NC) IBM SPSS 21 (SPSS Chicago, Ill., USA) and PROC StatXact for SAS, version 8 (Cytel Inc., Cambridge, MA 02139 USA, http://www.cytel.com) was used to compute statistics based on exact procedures.

Mann-Whitney test was used to calculate the difference in beta cell mass between T2DM organ donors with and without e islet antibodies or the DR3/DR4 class II allelic combination.

## Results

### Markedly Reduced Beta Cell Secretory Capacity in clinically diagnosed T2DM subjects with Islet Autoimmunity

**Figure 1:**
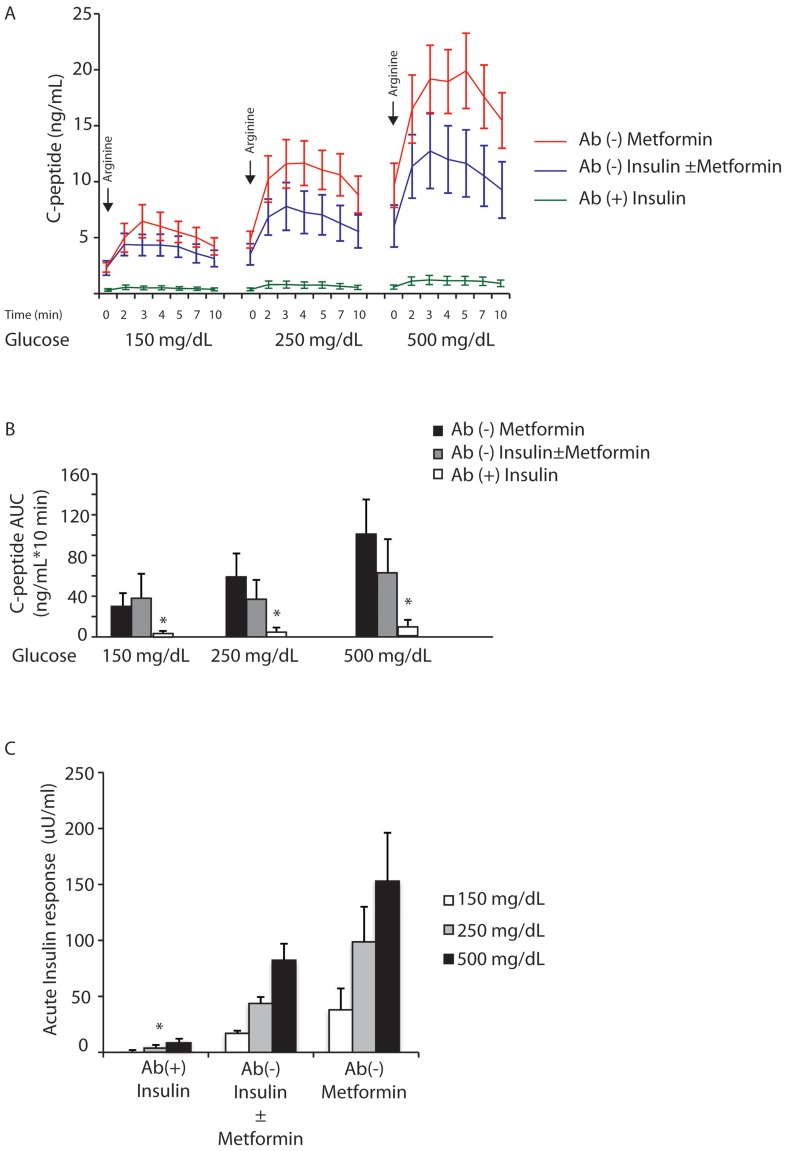
Acute C-peptide and insulin response after arginine and glucose infusion. **A**. Comparison of C-peptide response as a function of time and glucose/arginine stimulation for clinically diagnosed T2D patients antibody positive (insulin±metformin)(n = 7), antibody negative (insulin±metformin) (n = 6) and antibody negative on metformin only (n = 5). Pulses of arginine were administered after clamping plasma glucose at 150, 250 and 500 mg/dL. **B**. The area under the curve (AUC) for C-peptide was calculated for the three groups. *p-value<0.0001 for antibody positive *vs*. antibody negative groups for all three glucose levels. Error bars indicate SEM. **C**. Comparison of acute insulin response (AIR) to arginine at three different plasma glucose levels (150, 250, 500 mg/dL) in antibody positive insulin therapy (n = 7), antibody negatives on insulin ± metformin (n = 6) and antibody negativesmetformin only (n = 5). *p-value<0.05 for antibody positive *vs*. antibody negative groups for all three glucose levels. Error bars indicate SEM.

The islet antibody positive group had significantly lower fasting C-peptide and pro-insulin levels as compared to the antibody negative groups (Table 1). Figure 1A shows the C-peptide levels during an arginine test at variable glucose levels (150, 250 and 500 mg/dL) according to previously established protocols [2]. These results demonstrate the existence of poor beta cell secretion in the antibody positive group for all time points. The antibody negative group on insulin±metformin demonstrated a more impaired C-peptide response to arginine when compared to the metformin only group. Multiple comparison of AUC of serum C-peptide levels measured during the arginine test at variable glucose levels (150, 250 and 500 mg/dL) confirmed a severe impairment of insulin secretion in the antibody positive group as compared to both antibody negative groups (p = 0.0001; p = 0.0001; p = 0.0001 respectively) (Figure 1B).

We then evaluated acute insulin responses to arginine (AIR) after clamping plasma glucose at 120, 250 and 500 mg/dL [2]. The presence of islet antibodies correlated with severely impaired β-cell function as demonstrated by a remarkably low AIR_max_ compared to the antibody negative groups (either subjects on insulin and/or metformin or metformin alone). For each group of patients (antibody positive on insulin±metformin, antibody negative on insulin±metformin and antibody negative on metformin) AIR values for different glucose levels were statistically significant (p = 0.012, 0.025, 0.015) respectively (Figure 1C). These p values compare antibody positive *vs*. antibody negative groups for all three glucose levels (120, 250 and 500 mg/dL).The group on metformin only had the highest stimulated insulin response at all glucose levels. Thus, these results demonstrate that the presence of positive islet autoantibodies in patients with a clinical diagnosis of type 2 diabetes identifies a group of patients with poor beta cell function.

### Markedly Diminished Beta Cell Mass in clinically diagnosed T2DM subjects with Islet Autoimmunity or the DR3/DR4 class II HLA allelic combination

Based on islet autoantibody testing and HLA genotyping performed on cadaveric organ donors in whom a pre-mortem clinical diagnosis of T2DM was made, we defined the following two groups of subjects: a) cadaveric donors with and without islet antibodies; b) cadaveric donors with or without high-risk HLA class II genotypes (Table 2). In particular, the first group consisted of two GAD65 autoantibody positive subjects (with age of onset of 23.3 and 30.9 years) and the second group of two antibody negative subjects carrying the T1DM high-risk alleles DR3/DR4 (with age of onset of 26.3 and 34.8 years). The definition of autoimmune diabetes in those subjects carrying the DR3/DR4 allele was based on the most recent ADA clinical practice recommendations on the diagnosis and classification of diabetes mellitus [30].

**Figure 2:**
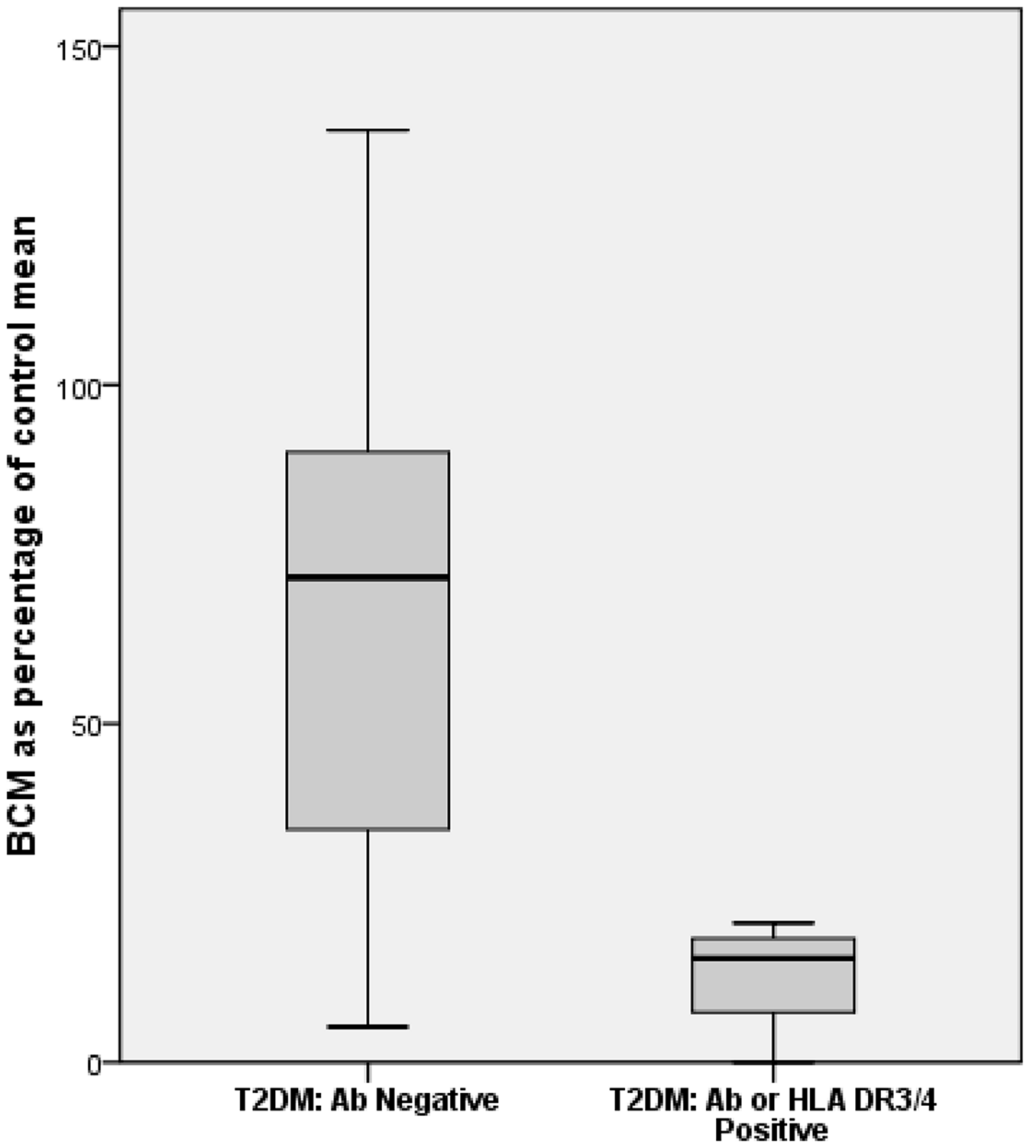
Beta cell mass determination. Beta cell mass (expressed as percentage of mean of donors without diabetes mellitus) in T2DM without autoimmune markers [mean 67.70±SEM 13.58 (n = 11) and T2DM with autoimmune markers (mean 12.80±SEM 4.54 (n = 4)] (p = 0.05) Amongst the donors with markers of autoimmune diabetes 4 out of 4 expressed pattern A pathology while among the donors without autoimmune markers, 3 out of 11 expressed pattern B pathology (p = 0.026).

**Figure 3:**
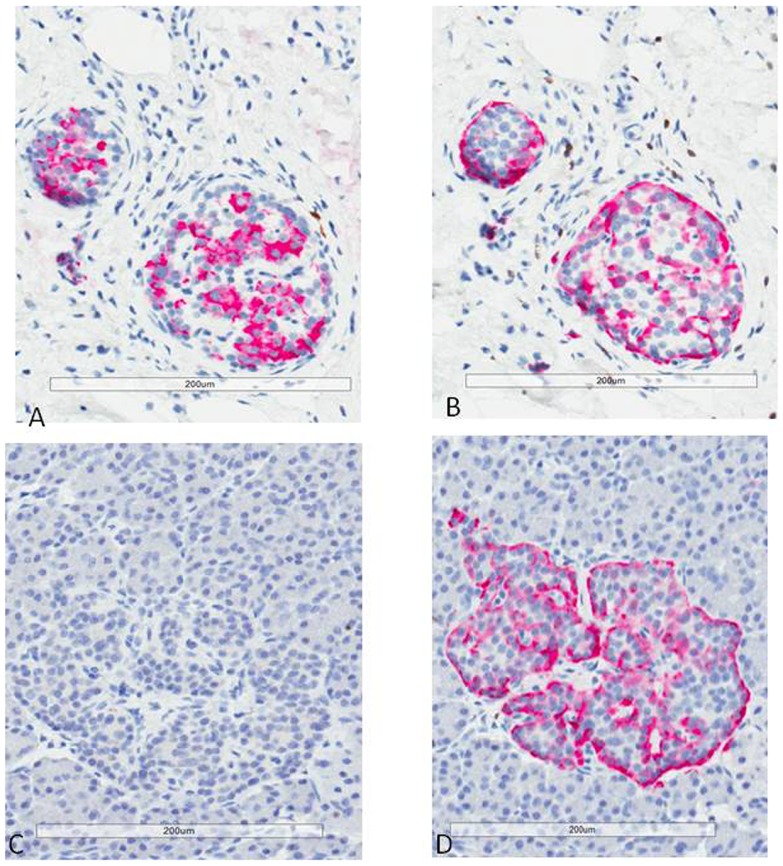
Insulin and glucagon immunostaining. Immunohistochemical staining for insulin (Panel A and C) and glucagon (Panel B and D) in two different islets from different area of the pancreas in a donor with T2DM and pattern A pancreatic pathology. The first islet (panel A and B) contains both insulin and glucagon positive cells, while the second islet (Panel C and D) contains only glucagon positive cells.

Quantification of beta cell mass in control donors revealed average BCM of 0.47 gms (median = 0.33 gms, range = 0.07 to 2.09 gms), while in the T2DM group the mean beta cell mass was 0.26 gms (median = 0.26 gms, range 0 to 0.65 gms). Type 2 diabetic donors with surrogate markers of autoimmune diabetes had reduced beta cell mass when compared to T2DM donors with no evidence of autoimmune diabetes (P = 0.05) (Figure 2). There was no significant difference in diabetes duration between these two groups. Amongst the donors diagnosed with T2DM who had autoimmune abnormalities (either islet autoantibodies or high-risk HLA class II alleles), 4 out of 4 exhibited pattern A pathology while among donors with T2DM and no evidence of autoimmune diabetes, 3 out of 11 exhibited pattern B pathology (P = 0.026) (Figure 3).

### Insulin Resistance is present in clinically diagnosed T2DM subjects with evidence for Islet Autoimmunity

These participants were also evaluated by a glucose clamp study. Disposition index (DI), which is defined as the ratio of insulin secretion over insulin sensitivity, was lower for the antibody positive group as compared to the other two groups, consistent with a defective beta cell function (p = 0.01 and 0.003 respectively) (Figure S1A). Whole body insulin-mediated glucose uptake as measured by the glucose infusion rate (GIR) was not significantly different between the three groups (Figure S1B). There was a trend towards higher adiponectin levels in the antibody positive group (8819 vs 15877 µg/ml). No differences in CRP levels were identified (Table 1).

**Figure 4:**
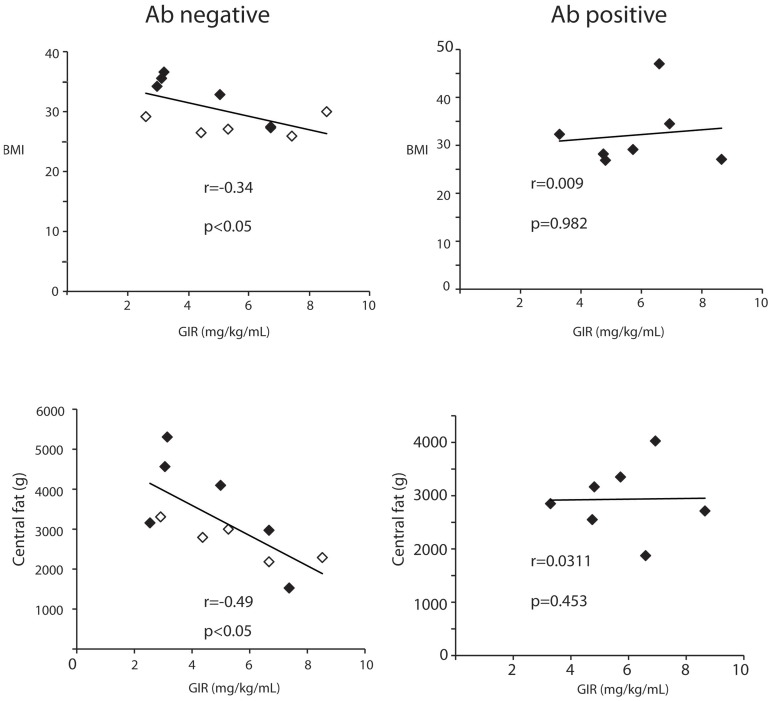
Insulin sensitivity in the setting of islet autoimmunity. Correlation between insulin sensitivity (GIR) and obesity (BMI, central fat) between all antibody negative (n = 11) and antibody positive on insulin±metformin (n = 7). Open squares indicate antibody negative on metformin only while close squares indicate patients on insulin±metformin.

It is known that there is a correlation between obesity, in particular central obesity, and insulin resistance [31]. As expected we found a negative correlation between BMI and GIR for the antibody negative group (Figure 4). Similar findings were seen when correlation between central fat and GIR was determined. Interestingly the antibody positive group demonstrated no correlation between GIR and BMI or GIR and central fat. These findings imply that in clinically diagnosed type 2 diabetics with autoantibodies against beta cells there is an obesity independent factor leading to insulin resistance.

In terms of lipid profile, the HDL levels were higher in the antibody positive group when compared to the antibody negative on insulin±metformin (74 vs. 41 mg/dL). There was a trend towards lower triglyceride levels in the antibody positive group (86 vs. 62 mg/dL). One subject in the antibody positive group had significantly higher fasting triglyceride levels (163 mg/dL) while the rest of the group had an average triglyceride level of 45 mg/dl (Figure S2).

## Discussion

The prevalence of both type 1 and type 2 diabetes has reached epidemic proportions worldwide and the understanding of the role of autoimmunity in diabetes has evolved along with the characterization of new disease phenotypes [32]. While the presence of autoantibodies directed to islet proteins is the hallmark of “classical” T1DM, a number of studies have shown that diabetes-related autoantibodies can be detected in 5–10% of patients with other types of diabetes, such as type 2 diabetes, that have not usually been associated with autoimmunity in their etiology (5, 7, 8). This phenotype seems to be different than T1DM in that the immunologic responses are mainly directed to GAD65 Ab and that there is a weaker association with HLA genotypes as compared to classical T1DM [8], [15]. A growing body of research has documented the case of patients who are generally adults, non-obese, who otherwise present a type-2 diabetes phenotype and who puzzlingly also have circulating islet autoantibodies. These characteristics have led to the term Latent Autoimmune Diabetes of Adults (LADA) [22]. Currently, the diagnosis of LADA relies primarily on the detection of autoantibodies against GAD65 in the serum of clinically diagnosed T2DM patients. In this regard, GAD65 testing provides the critical first identifier to detect this unique diabetic disease phenotype [13], [19], [22], [33]. Despite a wealth of data supporting the role of genetic factors in T1DM and T2DM, little is known regarding the genetics of LADA [22], [34]–[36]. Additionally, it has also been shown that, as is the case with “canonical” T1DM patients, LADA patients also possess T-cells reactive to islet proteins [37].

In the present study we characterized a group of patients clinically diagnosed as having T2DM with positive autoantibodies against islet autoantigens. Measurement of β cell function through arginine and glucose stimulation determined that these patients exhibited a significant impairment of β cell function when compared to a group of antibody negative of similar age, BMI and time of diabetes diagnosis. Furthermore, unlike the antibody negative group, patients with evidence of autoimmunity had insulin resistance that was independent of BMI or central obesity. Of note, previously reported observations indicate that psychosocial factors influence the risk of autoimmune diabetes in adults, possibly through mechanisms related to insulin resistance [38]. Hence, present compelling evidence to suggest that, despite high BMI, subjects with diabetes and islet autoantibodies exhibit severe beta cell dysfunction at baseline and in response to a potent insulin secretagogue (arginine). This impaired beta cell function may be caused by an autoimmune-mediated beta cell dysfunction/destruction with a loss of beta cell mass.

Quantification of beta cell function through arginine/glucose stimulation determined that as expected, individuals with a shorter time since diagnosis of diabetes and that required no insulin have relatively preserved beta cell function (metformin group only). This study demonstrates that the antibody positive T2DM patients exhibit a significant impaired beta cell function when compared to the other two groups. As a matter of fact, the magnitude of AIR_max_ is likely to decrease in those antibody positive diabetic patients such as those clinically diagnosed T2DM patients with evidence of islet cell autoimmunity described in this study.

A strength of the present work is that we evaluated all the nPOD pancreatic tissue from T2DM organ donors available through December 31, 2013 [25]; to the best of our knowledge, this is the largest collection of pancreatic tissue from T2DM cadaveric donors. However, a limitation of this study is a relatively small sample size. The previously discussed *in vivo* observations are reinforced by the analysis of pancreatic sections from T2DM organ donors. In particular, we determined that a subset of organ donors with T2DM showed pattern A pathology which is strongly associated with autoimmune diabetes. Interestingly, these donors were also positive for either islet autoantibodies or the autoimmune diabetes class II allelic combination DR3/DR4. The presence of pattern A pathology in T2DM in association with islet autoimmunity confirms that in subset of cases of T2DM, beta cell dysfunction and loss may well be related to autoimmune mechanisms [26]. It is not clear, at this stage, whether these individuals have a mixed diabetes with immune-mediated beta cell loss superimposed to other mechanisms causing insulin resistance or whether immune mechanisms are responsible for both beta cell loss and insulin resistance. In this small group, there was a somewhat reduced beta cell mass in islet antibody and HLA DR3/DR4 positive donors suggesting a more pronounced beta cell loss in the presence of an autoimmune component to the disease.

Of note, a subanalysis from the ADOPT trial characterized GAD Ab positive T2DM patients [39]. Unlike our findings, this study suggested that GAD Ab positive and negative patients had similar beta cell function. The discordance in findings could be due to the fact that only patients that were drug naïve for up to 3 years were included in the ADOPT trial and the analysis was performed in early stages of the natural history of the disease, whereas the LADA patients evaluated in this study were all on insulin therapy and had at least a 5-year-duration of diabetes. In summary, we now present convincing evidence supporting that the presence of islet cell autoantibodies in a T2DM population defines a group of these subjects with significant impairment of beta cell function and beta cell mass.

Studies have shown a strong relationship between diabetes and inflammation [9], [40]–[42]. Inflammation and dysregulated adipokine secretion have been implicated in obesity-related insulin resistance and type 2 diabetes. C-reactive protein (CRP) is one of the most significant acute-phase proteins in humans and has been associated with an increased risk in the development of diabetes [43]. We previously reported [8] no significant difference in CRP levels between antibody positive or antibody negative individuals clinically diagnosed with T2DM. Adiponectin is regarded as an anti-inflammatory adipokine and as such usually correlates positively with insulin sensitivity. In T1DM adiponectin has been described as higher than that of non-diabetics [44]. These observations could not be explained by differences in age, gender or fat distribution. We found a trend towards higher adiponectin levels for the antibody positive group. This would support the hypothesis that factors unrelated to adiponectin may contribute to the development of insulin resistance in autoimmune diabetes.

The glucose clamp results revealed a good correlation between BMI and GIR as well as central fat and GIR in the antibody negative group. An interesting observation from this study is that this correlation is lost for patients with evidence of autoantibodies. These findings could suggest that a subgroup of patients, previously estimated to be in the 5–10% range of T2DM, has insulin resistance that is independent of obesity. Interestingly, obesity independent insulin resistance has also been described as present in type 1 diabetes [45]. It could be hypothesized that glucotoxicity might be playing a role in the development of insulin resistance in LADA. The overall glycemic control in our study was relatively adequate and not significantly different between the GAD65 Ab positive and negative groups as determined by HbA1c suggesting that glucotoxicity was not an important factor. There is gathering evidence to suggest that glucose variability may be important in the development of insulin resistance [46]. We could expect that significant beta cell dysfunction would be associated with a higher degree of glucose variability. This glucose variability has been associated with the development of oxidative stress and higher levels of advanced glycation end products [47]–[49]. In LADA these factors could be playing an important role in the development of insulin resistance regardless of obesity. Defining the impact of these mechanisms in the development of insulin resistance in this particular population is key as these same factors are involved in the development of a dysfunctional endothelium leading to cardiovascular morbidity and mortality [50].

In summary, our study provides compelling evidence to suggest a significant impairment of beta cell function and mass in clinically diagnosed T2DM with evidence of islet autoimmunity. This phenotype could be the end result of an immune-mediated beta cell destruction. This particular subgroup of patients seems to exhibit insulin resistance in a similar fashion of that of antibody negative T2DM and independent of BMI or central obesity. Routine islet autoantibody evaluation in all T2DM patients may identify a subgroup of LADA with potentially robust responses to immune therapy aimed at preserving insulin production and beta cell mass in the early stages of the natural history of LADA.

## Supporting Information

Figure S1 **A. Disposition index measurements.** Disposition Index (DI: β-cell compensation for insulin resistance) obtained from the glucose clamp studies. p-value<0.05 for antibody negative on metformin *vs*. antibody positive on insulin±metformin. Horizontal bars indicate mean values. **B**. Comparison of Glucose Infusion Rate (GIR) obtained during the glucose clamp studies as measurement of insulin sensitivity. No significant difference was found between groups.
(PPTX)

Figure S2 **Triglyceride levels.** Comparison of fasting triglyceride levels between the clinically diagnosed T2D patients antibody positive on insulin±metformin, antibody negative on insulin±metformin and antibody negative on metformin. Horizontal bars indicate mean values.
(PPT)
