# Supplementary figures and images for: Islet Autoimmunity Identifies a Unique Pattern of Impaired Pancreatic Beta-Cell Function, Markedly Reduced Pancreatic Beta Cell Mass and Insulin Resistance in Clinically Diagnosed Type 2 Diabetes
Source: PLoS One. 2014 Sep 16;9(9):e106537. doi: 10.1371/journal.pone.0106537 (PMC4165581; doi:10.1371/journal.pone.0106537)

## Slide 1
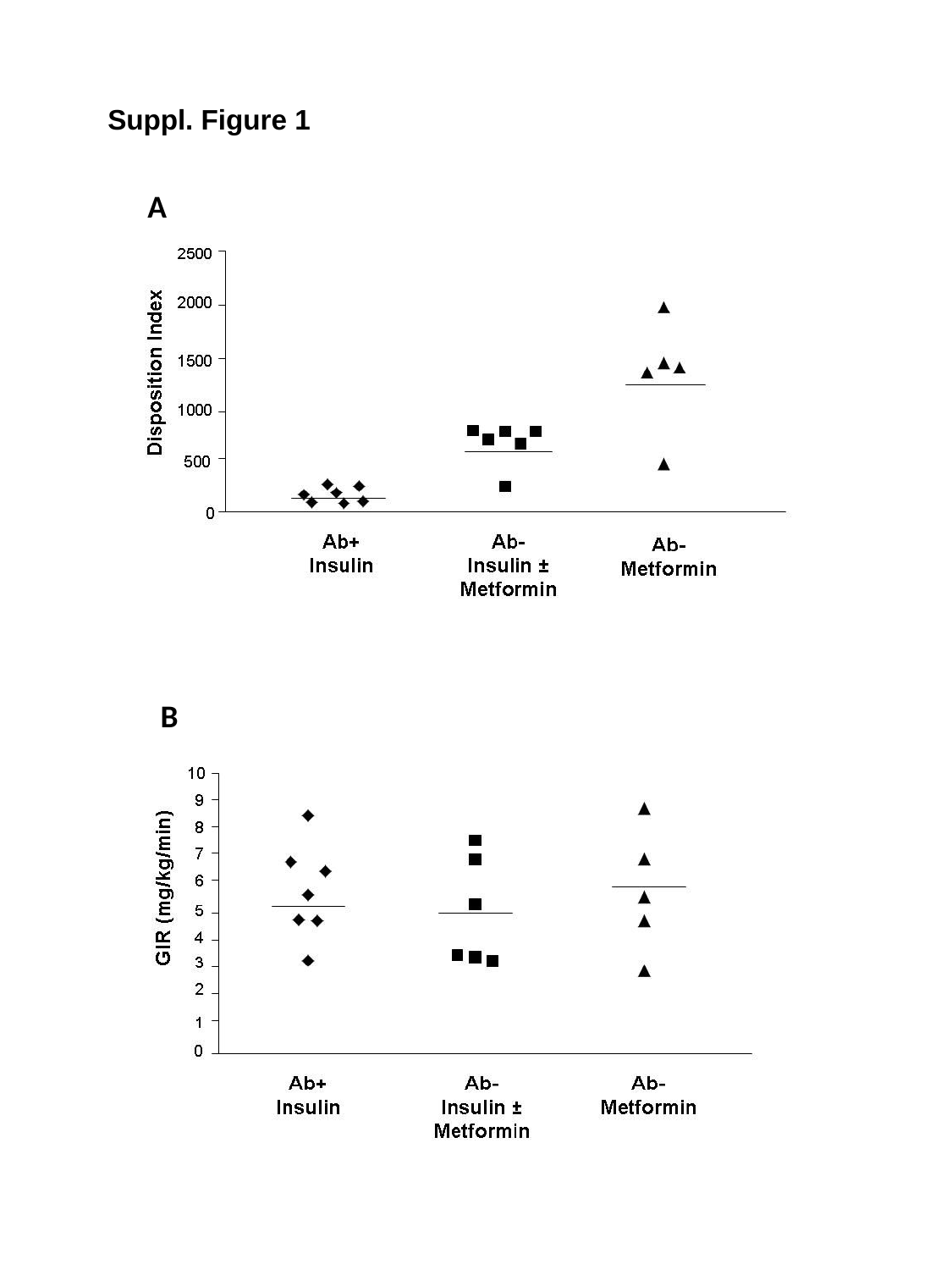

Suppl. Figure 1
A
B

Supplement: Figure S1 — A. Disposition index measurements. Disposition Index (DI: β-cell compensation for insulin resistance) obtained from the glucose clamp studies. p-value<0.05 for antibody negative on metformin vs. antibody positive on insulin±metformin. Horizontal bars indicate mean values. B. Comparison of Glucose Infusion Rate (GIR) obtained during the glucose clamp studies as measurement of insulin sensitivity. No significant difference was found between groups. (PPTX) [file pone.0106537.s001.pptx]

## Slide 1
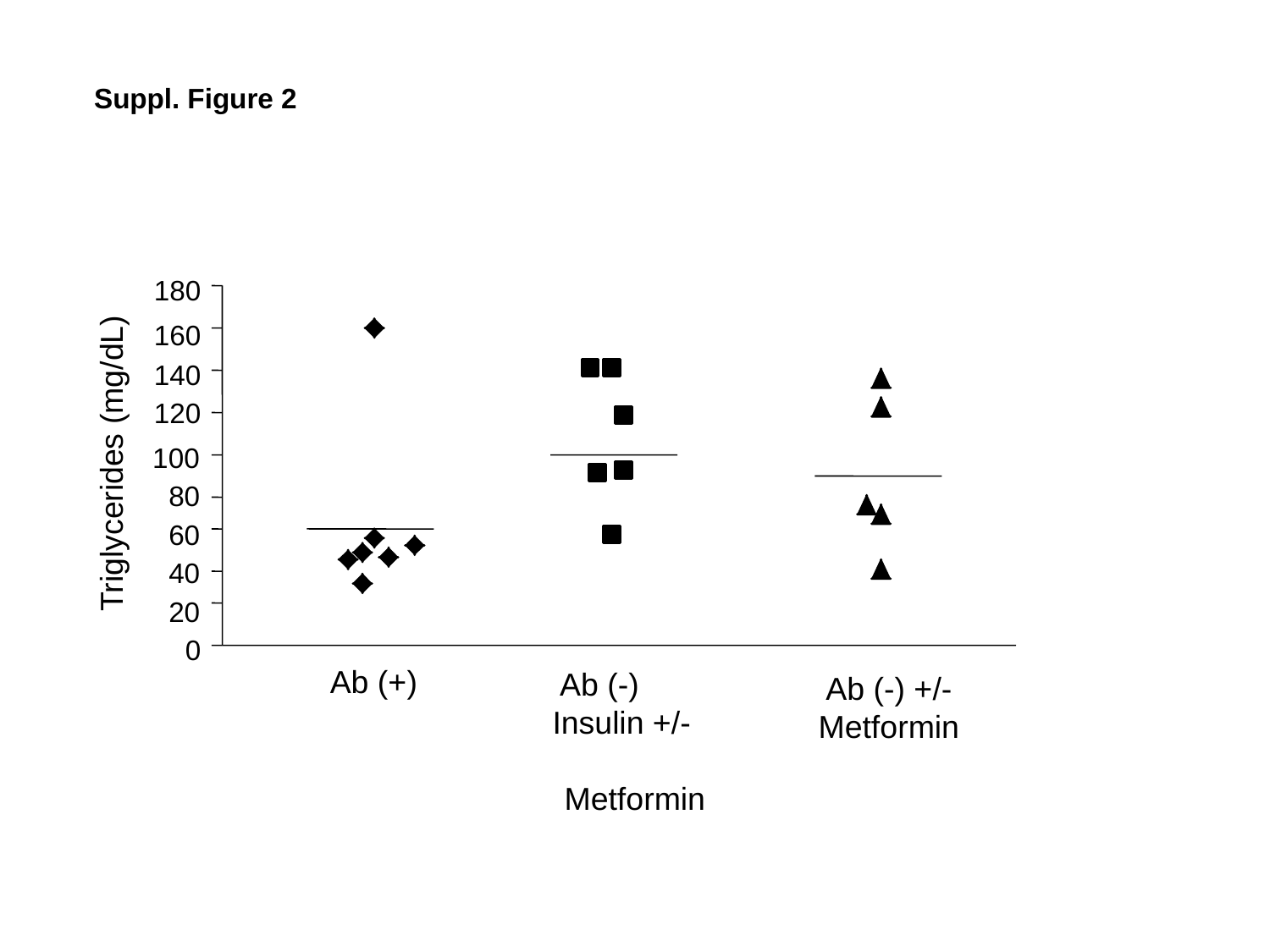

Suppl. Figure 2
180
160
140
120
Triglycerides (mg/dL)
100
80
60
40
20
0
Ab (+)
Ab (-) Insulin +/- Metformin
Ab (-) +/- Metformin

Supplement: Figure S2 — Triglyceride levels. Comparison of fasting triglyceride levels between the clinically diagnosed T2D patients antibody positive on insulin±metformin, antibody negative on insulin±metformin and antibody negative on metformin. Horizontal bars indicate mean values. (PPT) [file pone.0106537.s002.ppt]
